# Analysis of Gene Expression Profiles Regulating Phenotypic Transformation of Vascular Smooth Muscle Cells by Endothelial Cell-Derived Exosomes

**DOI:** 10.33549/physiolres.935541

**Published:** 2025-08-01

**Authors:** Tao KANG, Xiaoqiang LI, Song HAN, Yaoliang LU

**Affiliations:** 1 Department of Vascular Surgery, The Second Affiliated Hospital of Soochow University, Suzhou city, Jiangsu province, China; 2 Department of Vascular Surgery, The Taicang Affiliated Hospital of Soochow University, Suzhou city, Jiangsu province, China

**Keywords:** Endothelial Cells, Vascular Smooth Muscle Cells, Exosomes, Long non-coding RNA, Circular RNA

## Abstract

To establish a co-culture cell model and implement high-throughput gene sequencing of exosomes, we preliminarily demonstrated that endothelial cell-derived exosomes play a role in modulating the phenotypic transformation of vascular smooth muscle cells (VSMCs) by means of differentially expressed long non-coding RNAs (lncRNAs) and circular RNAs (circRNAs). Primary rat aortic endothelial cells (ECs) and VSMCs were cultured for morphological observation, immunofluorescence (IF), and western blotting (WB). A co-culture model was established using a transwell system. A comparative analysis of α-smooth muscle actin (α-SM actin), a marker of the contractile phenotype, and vimentin, indicative of the synthetic phenotype, was conducted to assess the expression levels in both co-culture and control setups. Isolated exosomes were obtained using an exosome-specific isolation kit, followed by detailed characterization using transmission electron microscopy (TEM) for morphological assessment, nanoparticle tracking analysis (NTA) for size distribution, and WB for protein profiling. Primary aortic ECs were isolated, cultured, and characterized. In the Transwell co-culture model, VSMCs transitioned to a contractile phenotype, exhibiting increased alpha-smooth muscle actin (α-SMA, contractile marker) and decreased Vimentin (synthetic marker). Exosomes were extracted, purified, and characterized by their morphology, diameter, concentration, and marker proteins (CD9, CD63, and CD81). RNA-seq and bioinformatic analyses were conducted on muscle cells before and after treatment. The Transwell-based ECs-VSMCs co-culture model significantly upregulates contractile phenotype protein expression in VSMCs, promoting their transition to a contractile state. Differentially expressed exosomal genes, including lncRNAs and circRNAs, modulate proliferation, differentiation, and phenotypic transformation of VSMCs.

## Introduction

Atherosclerosis, a primary contributor to heart attacks, strokes, and restenosis within stents, significantly endangers human well-being. When blood vessels are damaged, endothelial cells (ECs) secrete cytokines, initiating a cascade of events in vascular smooth muscle cells (VSMCs). These events include phenotypic changes, proliferation, migration, extracellular matrix remodeling, and other pathological alterations that ultimately lead to atherosclerosis [1,2]. Phenotypic shifts in VSMCs play pivotal roles in vascular remodeling. Consequently, inhibition of VSMCs transformation has emerged as a novel approach for combating atherosclerosis. Most previous studies have relied on isolated cell cultures, which fail to replicate the complex environments found in living organisms. In contrast, co-culturing different cell types more accurately simulates *in vivo* conditions and elucidates intercellular interactions. Thus, culturing ECs alongside VSMCs provides an optimal in vitro model for simulating the physiological state of blood vessels and examining cell-to-cell interactions.

In this study, we established a co-culture system utilizing a Transwell, incorporating aortic ECs and VSMCs. Western blotting (WB) analysis was employed to assess the expression levels of proteins that serve as markers for both the contractile and synthetic phenotypes within VSMCs. An exosome kit was used to isolate exosomes, and their morphology was examined using transmission electron microscopy (TEM). A Malvern nanoparticle size analyzer was used to measure exosome diameter and concentration. WB analysis confirmed successful exosome isolation with sufficient purity through the detection of exosome marker proteins CD9, CD63, and CD81. The exosomes were subjected to high-throughput gene sequencing, followed by bioinformatic analysis of differential gene expression profiles to elucidate signaling pathways and downstream target genes involved in VSMCs phenotypic transformation. This study provides a foundation for subsequent investigations of the intercellular communication between ECs and VSMCs.

## Materials and Methods

### Experimental animals

A cohort of ten male Sprague Dawley rats, aged between 4 to 6 weeks with an average weight of 80 grams (±6.8 g), was sourced from the Shanghai Laboratory Animal Center. These animals were certified to be specific pathogen-free grade. All animal-based studies adhered to the guidelines set forth in the “People’s Republic of China Regulations on Experimental Animals Administration” and followed the “Soochow University Experimental Animals Management Regulations.” Ethical review and approval for our experiments were granted by the Animal Welfare and Ethics Committee of the Taicang Affiliated Hospital of Soochow University under the specific approval code 2019-DW-013. All experiments were conducted in triplicate to ensure reliability and reproducibility of the results.

### Key reagents and instruments

The complete medium for rat aortic ECs ( Procell Life Science & Technology Co., Ltd” (Wuhan, China, CM-R075)), it contained 5 % FBS, 50ng/mL VEGF, 10ng/mL EGF, 20ng/mL bFGF and 1 % penicillin and streptomycin,CD9, CD31, CD63, and CD81 monoclonal antibodies (Abcam, USA), fluorescein (Cy3)-labeled goat anti-rabbit IgG (Boster, Wuhan, China), exosome extraction kit (exoEasy Maxi Kit, Qiagen), GW4869 exosome inhibitor (Yensen, China), SW-CJ-IF clean bench, constant temperature CO_2_ incubator (Thermo, USA), 6-well Transwell culture plate (0.4 mm) (Costar 3450, USA), Olympus inverted microscope, fluorescence microscope, Agilent 2100 Bioanalyzer (Agilent Technologies, USA), and NovaSeq 6000 (Illumina, USA) were employed in this investigation.

#### Primary cell culture and identification

##### Isolation, culture, and passaging of rat aortic endothelial cells

Primary Aortic ECs were isolated from 10 Sprague-Dawley rats and three independent experiments were conducted for each experimental group. Sprague Dawley rats were euthanized by cervical dislocation, followed by sterilization of the skin on the ventral side. The abdominal cavity was systematically opened in layers under a sterile laminar flow hood and the aorta was excised and bisected longitudinally. The intima was positioned with the luminal surface facing downward in a Petri dish, and trypsin was introduced for enzymatic digestion. The dishes were placed in a culture incubator. Using fine microscope forceps, the intimal surface underwent gentle, repeated abrasion. The resulting solution was collected using a pipette and filtered through a cell strainer. Following centrifugation, the cell suspension was prepared and transferred to culture flasks. The cells were resuspended in EC medium and incubated for cultivation. The medium was performed every 2–3 days, with daily observations and documentation of cell morphology using an inverted microscope.

##### Identification of rat aortic endothelial cells

ECs were initially plated in culture dishes to promote cell adhesion, followed by fixation with a 4 % paraformaldehyde solution. Subsequently, the cells were treated with 0.5 % Triton X-100 for permeabilization and all procedures were performed at ambient temperature. After rinsing with phosphate-buffered saline (PBS), the cells were blocked with goat serum, followed by the application of CD31 primary antibody. Incubation of the samples was performed at a temperature of 4°C in a moist atmosphere overnight. On the subsequent day, the slides underwent a washing process with PBS solution. Subsequently, a solution of goat anti-rabbit IgG labeled with the fluorescent dye Cy3 was introduced. This was followed by further incubation at 37 °C in a chamber maintained at a high humidity. Cell nuclei were stained with DAPI under low-light conditions. Subsequent to the last rinse with PBS, the slides were secured for observation and image capture using a fluorescence microscope.

##### Expansion, culture, and passaging of aortic smooth muscle cells

The A7r5 cell strain, which consists of VSMCs from the thoracic aorta of rats, was sourced from the Shanghai Cell Repository of the Chinese Academy of Sciences. These cells were cultivated at 37 °C, and the culture medium was refreshed at intervals of 2–3 days. Routine passaging was conducted and cellular morphological characteristics were observed and documented daily using an inverted microscope.

##### Construction of transwell cell co-culture model

Group A: VSMCs cultured alone (blank control)
Group B: ECs-VSMCs co-culture
Group C: ECs-VSMCs co-culture with GW4869 exosome inhibitor (negative control)

Third-passage aortic ECs and VSMCs were diluted to a density of 1×10^6 cells per milliliter. Group A consisted of isolated VSMCs. Group B employed a 6-well Transwell plate, wherein ECs were cultured in the apical compartment, and VSMCs were placed in the basal compartment for co-culture. Group C incorporated GW4869 exosome inhibitor into the co-culture medium.

##### Western blot analysis of the expression of contractile phenotype marker proteins in vascular smooth muscle cells

Aortic VSMCs protein samples from the three experimental groups were analyzed via WB for α-SM actin and vimentin expression. Bicinchoninic acid (BCA) assay was implemented to quantify protein concentrations. The samples were then subjected to sodium dodecyl sulfate–polyacrylamide gel electrophoresis (SDS–PAGE) for protein separation, followed by transfer to a membrane. This was achieved by probing with primary and secondary antibodies. Subsequently, the blots were developed using a colorimetric method and analyzed using chemiluminescence for protein detection.

#### Isolation and identification of exosomes

##### Exosome extraction: exosomes were isolated using the exoeasy maxi kit in accordance with the manufacturer’s protocol

###### Exosome characterization

Nanoparticle tracking analysis (NTA): The isolated exosomes were analyzed using a Malvern nanoparticle size analyzer.

TEM Exosomes were prepared by washing with PBS and fixing with 2 % osmium tetroxide at 4°C, followed by PBS washing, ethanol gradient dehydration, embedding solution impregnation, embedding, uranyl acetate, lead citrate staining, and subsequent examination using a transmission electron microscope (Hitachi HT-7800).

WB Analysis Cell collection and lysis were performed, followed by retrieval of the supernatant fraction. Protein content was subsequently quantified using the BCA assay. Protein separation was achieved by SDS–PAGE, and the resolved proteins were blotted onto membranes. The membranes were then exposed to primary antibodies targeting CD9, CD63, and CD81, prior to incubation with the corresponding secondary antibodies. Subsequently, the membranes were fixed and developed.

#### Analysis of Differential Expression of long non-coding RNAs and Circular RNAs in Exosomes Using High-Throughput Gene Sequencing

##### Total RNA extraction, quality control, library construction, and DNA sequencing

Trizol reagent was used to isolate total RNA, which was then subjected to rRNA depletion using a Ribo-Zero rRNA Removal Kit. RNA was subsequently processed into sequencing libraries using a TruSeq kit for stranded total RNA library preparation. Library quality and quantification were assessed using BioAnalyzer 2100. Paired-end sequencing encompassing 150 cycles was performed using the Illumina NovaSeq 6000 platform.

##### Data extraction and bioinformatics analysis

Following sequencing, paired-end reads were obtained and subjected to quality assessment using a Q30 threshold. The Cutadapt tool facilitates the elimination of adapter sequences and filtering of substandard reads, thereby generating a dataset enriched with high-fidelity reads[3]. The sequences were then mapped against the reference rat genome, specifically the UCSC RN5 assembly, employing hisat2 alignment tool. HTSeq software was utilized to generate raw transcript counts for the long non-coding RNAs (lncRNAs) expression profiles. Data normalization and computation of fold change and p-value across sample comparisons were conducted using the EdgeR computational tool. Differentially expressed lncRNAs and circular RNAs (circRNAs) were identified, followed by Gene Ontology (GO) and Kyoto Encyclopedia of Genes and Genomes (KEGG) pathway enrichment analysis. The putative target genes of lncRNAs and circRNAs were predicted based on their proximity, and GO and KEGG pathway enrichment analysis were subsequently conducted on these predicted target genes.

##### Statistical Analysis

Data entry and analysis were performed using the GraphPad Prism 7. The comparison between two distinct groups was performed using a t-test for independent samples, whereas to evaluate differences across multiple groups, a variance analysis (ANOVA) was implemented. Numerical data are depicted with the average value ± standard deviation (x ± s). The threshold for statistical significance was set at a p-value of less than 0.05.

## Results

### Morphological and immunofluorescence identification of cells

#### Morphological identification of aortic endothelial cells

**Fig. 1:**
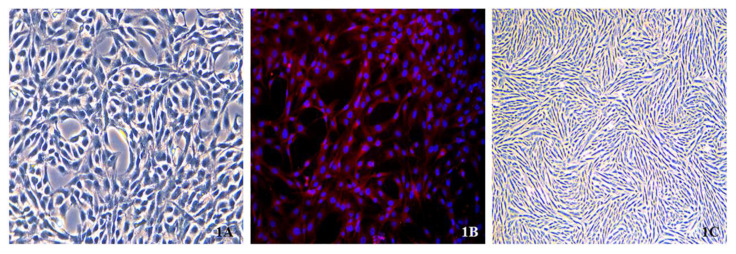
**A**) Primary aortic ECs were isolated from 10 Sprague-Dawley rats and three independent experiments were conducted for each experimental group. The fifth generation exhibited cobblestone morphology and lumen-like structures (200× objective magnification, scale bar=50μm). **B**) IF of aortic ECs with red fluorescence for CD31 (>90 % positivity) and blue for DAPI (200× objective magnification, scale bar=50μm). **C**) Second-generation aortic VSMCs displaying parallel alignment in single or multiple overlapping layers. To form ridge-and-valley patterns (200× objective magnification, scale bar=50μm).

After the initial inoculation, the cells exhibited a spherical morphology with heterogeneous dimensions. Within 48 h, they demonstrated substrate adherence, predominantly adopting a short spindle shape while also manifesting polygonal, spindle-like, and irregular morphologies. The colonies were characterized by an abundant cytoplasm and small nuclei. After two passages, the cells displayed increased uniformity, primarily assuming spindle-shaped or spindle-like forms, with some cells exhibiting stellate projections. Their nuclear-cytoplasmic boundaries were well defined, and numerous mitotic figures were observed. By the fifth passage, cells arranged in a cobblestone pattern formed lumen-like structures (Fig. 1A).

#### Immunofluorescence identification of aortic endothelial cells

Immunofluorescence (IF) detection of CD31 in rat aortic ECs (200X) showed over 90 % CD31 positivity, indicating a cell purity above 90 % (Fig. 1B).

#### Morphological observation of aortic smooth muscle cells

Twenty-four hours after revival, the cells began attaching to the substrate and displayed various morphologies such as elliptical, elongated spindle-shaped, triangular, and spindle-like forms, with centrally positioned ovoid nuclei. After 4–6 days, cellular fusion led to a predominantly elongated spindle shape, with abundant cytoplasm and dendritic projections. The cells were arranged in parallel monolayers or overlapping multilayers and exhibited a peak-and-valley growth pattern. At low densities, they formed a network structure, whereas at high densities, they organized into swirling or palisade configurations (Fig. 1C).

#### Western Blotting Analysis of Phenotypic Transition Marker Proteins in Aortic Smooth Muscle Cells

**Fig. 2:**
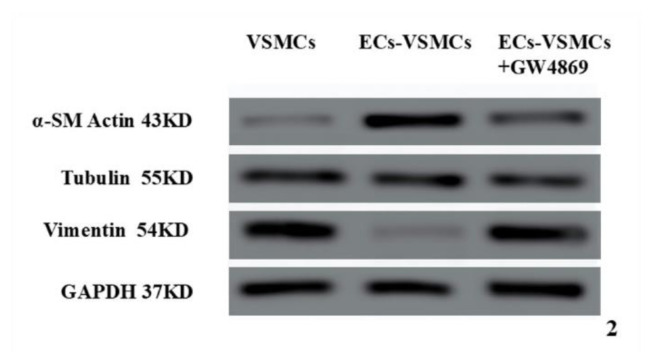
Comparison of α-SMA and Vimentin, contractile phenotypic markers of VSMCs in the aorta, across three conditions: VSMCs cultured alone group, ECs-VSMCs co-culture group, and ECs-VSMCs co-culture with GW4869 exosomal inhibition group.

The expression of α-SM actin(43KDa), a contractile phenotypic protein marker in aortic VSMCs, was significantly increased, whereas that of vimentin(54KDa), a synthetic phenotypic protein marker, was significantly decreased (Fig. 2).

#### Isolation and Identification of Endothelial Cell-Derived Exosomes

**Fig. 3:**
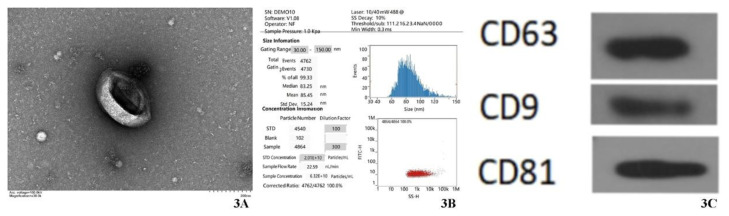
**A**) TEM (×30,000, voltage = 100.0 kV) reveals that ECs exosomes have a double-concave disc structure. **B**) NTA showed an average diameter of 85.45 nm, with 99.33 % of the exosomes at this size, consistent with typical extracellular vesicles (30–150 nm). **C**)WB analysis confirmed high expression of exosome marker proteins CD9, CD63, and CD81 in ECs exosomes.

Morphological characterization by TEM (Fig. 3A, scale bar: 200 nm) TEM revealed aortic EC-derived exosomes exhibiting classical cup-shaped (biconcave disk) morphology.

Particle size distribution analysis by NTA (Fig. 3B) NTA quantification demonstrated that the isolated extracellular vesicles (EVs) exhibited monodisperse size distribution with a mean particle diameter of 85.45 ± 15.24 nm (mean ± SD, n=3 independent isolations). Notably, 99.33 % of particles fell within the 30–150 nm range.

Protein marker validation by WB analysis (Fig. 3C) WB analysis confirmed the enrichment of exosomal transmembrane proteins CD9, CD63, and CD81 in the isolated vesicles.

### Analysis of Differential Expression Profile of Exosomal LncRNAs and CircRNAs Genes

#### Differentially Expressed Genes

Using the criteria of fold change (|log2Fold Change| > 2) and significance level (q<0.01), we identified differentially expressed genes in exosomes from the ECs-VSMCs co-culture group compared to VSMCs cultured alone. Our analysis revealed 4,276 differentially expressed genes, comprising 3,303 upregulated and 973 downregulated genes, demonstrating a statistically significant effect (P<0.05) (Table 1).

#### Gene Ontology (GO) Analysis of Differentially Expressed Genes

**Fig. 4:**
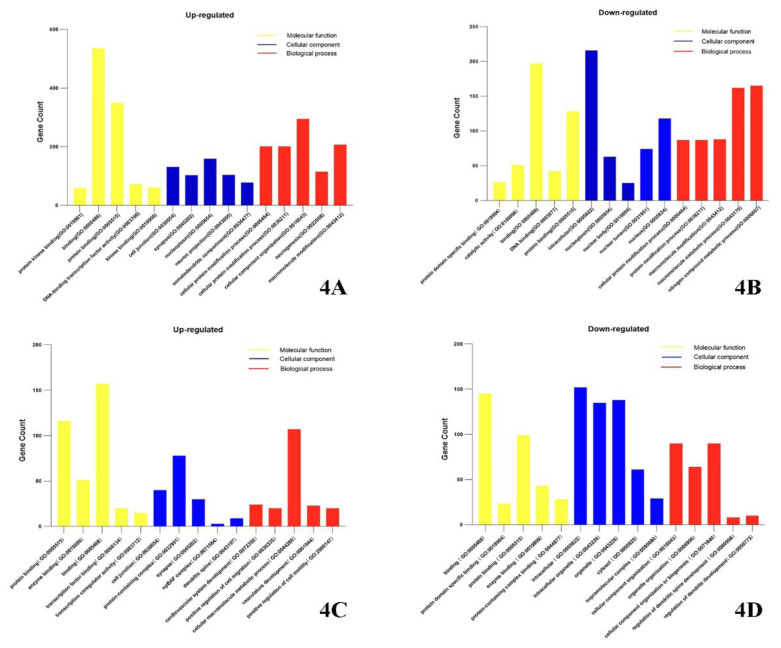
GO analysis of differentially expressed lncRNAs and circRNAs, with yellow indicating molecular functions, blue indicating cellular components, and red indicating biological processes. **A**) Upregulated lncRNAs; **B**) Downregulated lncRNAs; **C**) Upregulated circRNAs; **D**) Downregulated circRNAs.

GO analysis was performed to elucidate the functional roles of differentially expressed lncRNAs and circRNAs. In comparison with the VSMCs control group, 1117 biological processes, 139 molecular functions, and 164 cellular components linked to differentially expressed lncRNAs were identified in the ECs-VSMCs co-culturegroup. These lncRNAs were enriched in processes such as protein binding, enzyme binding, transcription factor activity, and enzyme activity, and were localized in the extracellular matrix, cell surface, and extracellular region of the plasma membrane, suggesting their roles in protein modification, transmembrane transport, localization, and biological development. For circRNAs, 669 biological processes, 72 cellular components, and 95 molecular functions related to differentially expressed circRNAs were identified in the ECs-VSMCs co-culture group. These circRNAs were notably overrepresented in pathways involving vascular system development, positive regulation of cell migration, and macromolecular metabolic processes and were localized at cell junctions, protein complexes, and actin cytoskeletons, with functions involving protein binding, enzyme binding, and transcription factor binding (Fig. 4).

#### KEGG Pathway enrichment Analysis of Differentially Expressed Genes

Enrichment analysis of KEGG pathways was conducted to compare differentially expressed lncRNAs and circRNAs between VSMCs cultured alone and the EC-VSMC co-culture group. These lncRNAs were enriched in 42 pathways, predominantly cellular senescence, endocytosis, phospholipase D signaling, phosphatidylinositol signaling, estrogen signaling, and MAPK signaling, with the highest enrichment observed for endocytosis. Similarly, differentially expressed circRNAs were enriched in 53 pathways, primarily the ErbB, Ras, PI3K-Akt, phospholipase D, and chemokine signaling pathways **(**Table 2).

## Discussion

Atherosclerosis, a condition of chronic oxidative inflammation within the vasculature, is characterized by the development of plaques due to lipid accumulation, which, in turn, induces arterial narrowing and potential blockage. Lipid accumulation induces EC dysfunction and leukocyte recruitment, resulting in phenotypic transformation of VSMCs. Predominantly composed of ECs and VSMCs, the vascular wall relies on the interplay between these cells to ensure the integrity and efficient functioning of mature vasculature [4]. Elucidating ECs and VSMCs interaction pathways during vascular remodeling can inform strategies for vascular disease prevention. VSMC phenotypic transition-induced arterial remodeling is a fundamental feature of vascular diseases. Investigating the factors and pathways that regulate this process could lead to the development of innovative preventive and therapeutic approaches for vascular proliferative disorders.

In our study, we selected primary rat aortic ECs and the A7r5 aortic smooth muscle cell line as our research subjects. We followed established methodological protocols [5,6] to isolate ECs by scraping the intima rather than using simple enzymatic release of the endothelium. Although this method may introduce contamination, we ensured the purity of ECs through rigorous screening, including microscopic removal of adventitial tissues. ECs were harvested at 80–90 % confluence, exhibiting typical cobblestone morphology (Fig. 1A) and high CD31 expression (>90 % positivity) (Fig. 1B), which preliminarily confirmed the high purity of ECs in the system. We acknowledge that CD31 is one of the most commonly used markers for EC differentiation. However, its expression in macrophages and platelets necessitates its combination with additional markers to enhance the specificity of EC identification. In future experiments, we will incorporate detection of von Willebrand factor (vWF) and vascular endothelial cadherin (VE-cadherin) to improve the accuracy of EC identification.

Additionally, we selected the A7r5 cell line rather than primary VSMCs as a model for VSMCs because of its robust proliferative capacity and phenotypic plasticity, which facilitates experimental manipulation and reproducibility of results. The A7r5 cell line has been extensively utilized in VSMC phenotype researches [7,8], demonstrating high experimental reproducibility and stable expression of fundamental VSMC markers including Alpha-Smooth Muscle Actin (α-SMA). However, we acknowledge that primary VSMCs may better recapitulate *in vivo* physiological conditions; their inherent limitations under standard experimental conditions, notably the propensity for rapid dedifferentiation in culture, necessitated the use of this established cell line to ensure experimental consistency and phenotypic stability throughout our investigations.

Additionally, Pioneering studies by Owens *et al*. [9] have established that the SRF-MYOCD transcriptional axis critically regulates the contractile phenotype of VSMCs, identifying α-SMA as a core molecular marker of differentiated VSMCs. Subsequent genetic perturbation experiments by Rensen *et al*. demonstrated that α-SMA deficiency directly impairs VSMC contractile function, evidenced by 72 % reduced vasoconstrictive capacity in knockdown models [10]. While these findings underscore α-SMA’s pivotal role, we acknowledge the inherent limitations of relying solely on α-SMA as a phenotypic marker in our current investigation. To enhance phenotypic resolution, future experiments will implement an optimized multi-marker detection protocol incorporating additional VSMC-specific markers, such as SM1 and SM2 myosin and calponin-1, to more accurately reflect the phenotypic transition of VSMCs [9].

In vitro studies on vascular pathophysiology have investigated ECs and VSMCs interactions, demonstrating that these interactions regulate paracrine molecule expression, thus necessitating an ECs and VSMCs co-culture system. Currently, the two-dimensional ECs-VSMCs co-culture model (Transwell) is widely utilized in vascular remodeling research. The study by San Sebastián-Jaraba *et al*. [11] developed a 3D co-culture model to explore the interactions between ECs and VSMCs and their impact on pathological vascular remodeling. This model mimics *in vivo* conditions and serves as an effective platform for studying intercellular communication between ECs and VSMCs. In our research, a Transwell-based two-dimensional co-culture system of aortic ECs and VSMCs was developed. This approach is technically simple and enables easy separation of distinct, homogeneous cell types without the need for cell sorting, thus laying the groundwork for further investigation.

Studies have demonstrated that EVs play a role in atherothrombosis by influencing endothelial function, lipid and leukocyte accumulation, plaque stabilization, and thrombosis [12,13]. EC-derived EVs upregulate vascular cell adhesion molecule-1 (VCAM-1) expression and facilitate leukocyte binding to VSMCs, whereas EVs originating from VSMCs fail to induce equivalent responses in ECs. EC-derived EVs also enhance signaling pathways that affect the VSMC phenotype [14]. This suggests that EC-derived EVs modulate the VSMCs phenotype. In this study, EC-derived exosomes were isolated using a commercial kit with tripartite validation compliant with MISEV2018 guidelines [15]: 1.TEM revealed a characteristic cup-shaped morphology (Figure 3A) attributable to dehydration artifacts during negative staining, fulfilling the ISEV quality criteria for small EVs [15]. 2. NTA demonstrated a monodisperse size distribution (mean diameter: 85.45 ± 15.24 nm; 99.33 % within 30–150 nm; Figure 3B), aligning with the size range of atheroprotective EC-derived exosomes reported by Hergenreider *et al*. [16]. 3.WB analysis confirmed the enrichment of tetraspanins CD9, CD63, and CD81 in exosomal fractions (Figure 3C), which confirmed their origin from multivesicular bodies (MVBs) [15]. The rigorous workflow ensures the functional integrity of the isolates for studying EC-VSMC crosstalk, particularly in atherosclerosis, where CD9+/CD81+ exosomes (~85 nm) regulate VSMC phenotypic switching through miR-143/145 delivery [16], a mechanism conserved across vascular pathologies [17].Whole-genome sequencing and high-throughput RNA-seq technologies have accelerated the discovery of diverse RNA species. EVs carry microRNAs, lncRNAs, and circRNAs, which regulate critical vascular cell processes such as differentiation, migration, proliferation, and apoptosis. These RNAs exert regulatory effects through targeting downstream genes at epigenetic, transcriptional, post-transcriptional, and translational levels [18]. For instance, lncRNA HCG11 influences VSMC proliferation and apoptosis via the miR-144-3p/FOXF1 axis[19]. The lncRNA ANRIL functions as a platform for WDR5 and HDAC3 complexes, facilitating phenotypic alterations in VSMCs [20]. Circ-SATB2 regulates VSMC differentiation, proliferation, apoptosis, and migration by upregulating STIM1, an miR-939 target gene, and inhibiting SM22α expression[21]. Exosomal circSHKBP1 orchestrates the miR-582-3p/HUR/VEGF axis, shields HSP90 from degradation, and fosters tumor advancement[22].

In our study, a co-culture system of aortic ECs and VSMCs was established to detect genes with markedly distinct expression patterns within the exosomes generated. High-throughput sequencing coupled with bioinformatics was utilized to scrutinize lncRNAs and circRNAs within exosomes derived from both the ECs-VSMCs co-culture and VSMCs alone control groups. This comparative study revealed notable disparities, aiming to decipher the regulatory roles of these genes in cellular processes and signaling, and shedding light on the molecular underpinnings of VSMC phenotypic transformation.

Compared to VSMCs control group, the ECs-VSMCs co-culture group exhibited significantly differentially expressed lncRNAs and circRNAs in their exosomes. Specifically, 3457 lncRNAs exhibited increased expression, while 1118 demonstrated decreased expression (P<0.05, log2 fold change ≥2). These lncRNAs orchestrate a range of biological processes, molecular functions, and cellular components, showing substantial overrepresentation of functions such as protein binding, enzyme binding, and processes such as protein modification and transmembrane transport. KEGG pathway analysis revealed enrichment in 42 signaling pathways, notably the endocytic pathway. Furthermore, Among the 682 circRNAs detected, 412 showed elevated expression levels and 270 exhibited reduced expression levels. These circRNAs modulate a variety of biological processes, cellular components, and molecular functions, with notable overrepresentation in key processes, including vascular system development and cell migration; cellular components, including cell junctions and protein complexes; and functions, such as protein and enzyme binding. KEGG analysis indicated enrichment in 53 signaling pathways, particularly the ErbB and Ras signaling pathways.

Studies by Boon *et al*. [23] and Hergenreider *et al*. [16] have demonstrated that differentially expressed lncRNAs and circRNAs can influence the phenotypic switching of VSMCs and arteriosclerosis progression by regulating various biological processes, molecular functions, and cellular components. Specifically, lncRNAs may exert their effects via the endocytosis signaling pathway, as exemplified by lncRNA H19 modulating VSMC migration through clathrin-mediated endocytosis in atherosclerosis [23], whereas circRNAs may modulate the phenotypic transition of VSMCs by sequestering key microRNAs (miRNAs) in the ErbB signaling pathway, akin to CircWDR77 acting as a miR-124 sponge to activate EGFR/ERBB2 signaling in neointimal hyperplasia [16].

## Study limitations

First, the current paradigm’s inability to discriminate exosomes from proliferative versus mature ECs, due to CD31’s pan-endothelial expression lacking stage-specific resolution, represents a key limitation. Future investigations should implement a tripartite isolation strategy:

1. FACS-sorted EC subpopulations (EdU+/Ki67+ proliferative vs. vWF+/VE-cadherin+ mature);
2. Stage-validated exosome harvest (CD63+/CD81+/Annexin V−);
3. Functional benchmarking through endothelial nitric oxide synthase (eNOS) and Matrigel tubule-genesis assays.

Second, our current study preliminarily characterized exosomes from monocultured ECs and EC-VSMC co-cultures. Subsequent investigations will implement a three-arm comparative design: monocultured ECs, monocultured VSMCs, and EC-VSMC co-cultures. This tripartite paradigm will systematically evaluate coculture-derived exosomes’ capacity to recapitulate pathophysiological crosstalk through dual RNA-seq profiling and functional validation in 3D vascular organoids.

Third, although significantly differentially expressed genes in exosomes from cell co-culture were identified and bioinformatics was utilized for initial predictions, PCR validation of target genes was absent, necessitating further research to elucidate the signaling pathways and regulatory mechanisms. Future studies should investigate molecular regulatory mechanisms in the vascular environment more comprehensively.

## Conclusion

In the co-culture setup, ECs interacted through exosomal exchange, promoting VSMCs transition to a contractile state, which may result in the restructuring of blood vessels. Future studies should aim to clarify the molecular mechanisms of gene regulation within exosomes originating from ECs, potentially offering new perspectives for the management and therapy of proliferative vascular diseases.

## Figures and Tables

**Table 1:** Top 20 upregulated and downregulated genes of differentially expressed LncRNAs and CircRNAs between two groups; DEGs: differentially expressed gene.

DEGs	Genes	Gene Names
LncRNAs	Up-regulated	LOC103692580, LOC103693575, LOC102549148, Slc7a6, LOC102554165, Rgag4, LOC103691115, LOC100911864, LOC103693057, LOC102551474, AABR06024793.1, LOC103692558, LOC103693781, LOC103691721, LOC102553833, LOC102553663, LOC102556061, Rc3h1, LOC102556753, RGD1562140
	Down-regulated	AABR06089048.1, LOC102547925, Rn50_X_0564.1, AABR06024739.1, Abhd17a, LOC103692435, LOC103692947, Plekha6, LOC103692885, LOC102547398, AABR06028812.1, LOC103693913, AABR06038510.1, AABR06048498.1, AABR06097863.1, LOC102550858, AABR06104230.1, Dbf4, LOC102555690, LOC102551570
CircRNAs	Up-regulated	AY172581.24, Cenpw, Plec, Cldn10, Jak1, Arnt2, Pdgfd, Mmp19, LOC100912338, Wnt3, Sde2, RGD1562018, Add1, Ctbp2, Sgcz, Serpinh1, Meis2, Gabrb2, Agbl1, Arhgap26
	Down-regulated	Nudcd3, Falz, Rsu1, AY172581.24, Ube4a, Vat1, Rsu1, Smc5, Mt-nd5, Tlk2, Bnc2, Ripk3, Rin3, Rn7sl1, Ash1l, Dusp16, Cdc6, Grin2d, Zfp106, Svep1, Cenpk, Ash1l, Dusp16, Cdc6, Grin2d, Zfp106, Svep1

**Table 2:** KEGG pathway enrichment analysis of upregulated lncRNAs and circRNAs between the two groups revealed the top five signaling pathways and their associated target genes; DEGs: differentially expressed gene.

DEGs	Pathway ID	Definition	Genes
Lnc RNAs	rno04144	Endocytosis	ACTR3//AMPH//ARPC1A//ARPC3//CAV1//CHMP7//DNM3//EGFR//EPN2
	rno04218	Cellular senescence	AKT3//CACNA1D//CAPN2//CCNA2//CDKN2A//CDKN2B//E2F3//ETS1//HIPK2
	rno04070	Phosphatidylinositol Signaling System	DGKE//DGKG//DGKQ//INPP5A//ITPK1//PLCB4//PTEN//SYNJ2
	rno04010	MAPK signaling pathway	AKT3//ANGPT1//CACNA1D//CACNA2D3//EGFR//ERBB4//FGF8//GRB2
	rno04012	ErbB signaling pathway	AKT3//EGFR//ERBB4//GRB2//NRG1//NRG4//SOS2
Circ RNAs	rno04926	Relaxin signaling pathway	COL4A6//MAPK14//MMP2//PRKACB//SHC3//SOS1//SOS2
	rno04012	ErbB signaling pathway	EIF4EBP1//NRG2//PLCG1//SHC3//SOS1//SOS2
	rno04014	Ras signaling pathway	PDGFD//PLCG1//PRKACB//RASA1//RASAL2//SHC3//SOS1
	rno04151	PI3K-Akt signaling pathway	CDC37//COL4A6//EIF4B//EIF4EBP1//FN1//JAK1//PDGFD
	rno04390	Hippo signaling pathway	CTNNA3//PARD3//PPP2R2A//TEAD1//TGFBR2//WNT3
